# Comparative immunogenicity of mRNA-1273 and BNT162b2 SARS-CoV-2 bivalent booster vaccines in US nursing home residents

**DOI:** 10.1080/21645515.2026.2705661

**Published:** 2026-08-04

**Authors:** Olajide J. Olagunju, Oladayo A. Oyebanji, Debbie Keresztesy, Dennis Wilk, Evan Dickerson, Tiffany Wallace, Laurel Holland, Mike Payne, Ellen See, Chia Jung Li, Eunice Lim, Yasin Abul, Clare Nugent, Alejandro B. Balazs, Jürgen Bosch, Christopher L. King, Brigid Wilson, Stefan Gravenstein, David H. Canaday

**Affiliations:** a Division of Infectious Diseases and HIV Medicine, Case Western Reserve University School of Medicine, Cleveland, OH, USA; b Division of Geriatrics and Palliative Medicine, Rhode Island Hospital, Brown University Health, Providence, RI, USA; c Center for Global Health and Diseases, Case Western Reserve University, Cleveland, OH, USA; d Ragon Institute of MGH, MIT, and Harvard, Cambridge, MA, USA; e Division of Geriatrics and Palliative Medicine, Alpert Medical School of Brown University, Providence, RI, USA; f Center of Innovation in Transformative Health Systems Research to Improve Veteran Equity and Independence (THRIVE), Veterans Administration Medical Center, Providence, RI, USA; g Geriatric Research Education and Clinical Center, Louis Stokes Cleveland Department of Veterans Affairs Medical Center, Cleveland, OH, USA

**Keywords:** SARS-CoV-2, vaccines, COVID-19, long-term care, nursing home, immunogenicity, mRNA vaccine, immunization, immunology, prevention

## Abstract

Nursing home residents (NHRs) remain among the most vulnerable to severe outcomes from SARS-CoV-2 infection. While mRNA-1273 (Spikevax, Moderna) and BNT162b2 (Comirnaty, Pfizer-BioNTech) vaccines are widely used in this population, comparative data on their immunogenicity, particularly after bivalent boosters, remain limited. We conducted a longitudinal immunologic evaluation of U.S. NHRs who received either the mRNA-1273 or BNT162b2 bivalent vaccine. Serum samples were collected 10–30 d post-vaccination. Anti-spike IgG levels were measured using a Luminex bead-based assay, and neutralizing titers were assessed via pseudovirus neutralization. Comparative analyses of the titer distributions were performed using two-sided Wilcoxon rank-sum tests. Both vaccine groups demonstrated strong humoral responses to the ancestral Wuhan strain and Omicron BA.4/5 subvariants. Spike-binding antibody levels and neutralization titers were comparable between the mRNA −1273 and BNT162b2 vaccine formulations. Conclusions: Both mRNA-1273 and BNT162b2 mRNA vaccines elicit similarly robust humoral immunity in NHRs, supporting their interchangeable use in booster strategies. Our findings underscore the importance of timely booster administration over exact product selection in protecting this high-risk, immunosenescent population.

## Introduction

Older adult nursing home residents (NHRs) remain among the most vulnerable populations affected by SARS-CoV-2^1^ Despite the remarkable success of SARS-CoV-2 vaccine formulations in reducing severe disease and death, questions persist regarding the consistency and durability of vaccine-induced immunity in frail, immunosenescent individuals.^2–4^ NHRs account for a disproportionately high burden of COVID-19-related morbidity and mortality, even after vaccine rollout campaigns.^5–7^

The FDA authorized the two SARS-CoV-2 mRNA vaccines most widely deployed in U.S. nursing homes, mRNA-1273 (Moderna) and BNT162b2 (Pfizer-BioNTech), for emergency use in late 2020.^8,9^ To evaluate how vaccine platform differences translate to immune protection among frail older adults, we conducted an immunologic evaluation of bivalent mRNA-1273 and BNT162b2 in United States NHRs.

## Material and methods

### Study design and participants

This study is part of an ongoing longitudinal cohort designed to evaluate the immunologic response to SARS-CoV-2 vaccination in NHRs. Since the initiation of the national COVID-19 vaccination campaign in December 2020, we have systematically tracked immune responses following each vaccine dose in NHRs.

Electronic medical records review and interviews were conducted to obtain information on demographics, comorbidities, functional status, and the use of immunosuppressing medications. For this phase of the study, we evaluated immunologic responses to first doses of bivalent mRNA-1273 and BNT162b2 vaccines administered between September 2022 and May 2023 across 31 nursing homes in Ohio and Rhode Island. Blood samples were collected between 10 and 30 d following vaccination. One additional NH was excluded from the analysis because it introduced an imbalance across brands in prior doses and sex. We included this facility in a sensitive analysis of 32 NH presented in the supplement.

Anti-spike IgG antibody levels were quantified in serum using the bead-based Luminex immunoassay and samples were diluted as needed to stay in the range of the assay 0.25–7200 BAU/ml.^10,11^ The neutralizing antibody titers were measured using the pseudovirus neutralization assay as performed previously with a 50% pseudovirus neutralizing antibody titers (pNT50) with a lower and upper limit of detection of 1:2 and 1:8748.^10,11^

### Statistical analysis

We conducted a comparative analysis of humoral immune responses elicited by the mRNA-1273 and BNT162b2 vaccines. Anti-spike antibody levels and neutralizing titers against the Wuhan and BA.4/5 variants were log-transformed and displayed as boxplots to show the titer distributions. Given the frequency with which we observed neutralizing titers at the upper limit of detection, group differences were assessed using a two-tailed Wilcoxon rank-sum test. A *p*-value < .05 was considered statistically significant. All presented *p*-values are unadjusted. Not all samples were tested for all assays; and not all clinical data could be collected for all subjects. We presented all available data. No imputation methods were implemented for missing values, and count of missing values are presented in tables. All statistical analyses were performed using R software (version 4.4.2)

### Ethical approval

This study utilizing verbal consenting process received ethical approval from the WCG Institutional Review Board (Study number 1316159) as central IRB and reliant review by University Hospitals of Cleveland IRB (STUDY20211074) and Brown University IRB (STUDY00001059). Verbal informed consent was obtained from all participants or their legally authorized representatives before enrollment and documented by study staff according to the IRB approved protocol.

### Results

Characteristics of NHR who received mRNA-1273 or BNT162b2 are shown in Table 1. Participants in both groups were similar in age and sex distribution. The median age was 75 y (IQR, 66–83) in the mRNA-1273 group and 76 y (69–87) in the BNT162b2 group. Females comprised 60% of mRNA-1273 recipients and 58% of BNT162b2 recipients. Racial and ethnic composition differed modestly between groups, with a greater proportion of White non-Hispanic participants among BNT162b2 recipients and a higher proportion of Black non-Hispanic participants among mRNA-1273 recipients. Most participants had received multiple prior vaccine doses, with the majority receiving a fifth dose in both groups (87% mRNA-1273 vs. 81% BNT162b2). Median time from vaccination to blood collection was a few days shorter in the mRNA-1273 group (14 d^12–15^) than in the BNT162b2 group (19 d^13–19^).

**Table 1.** Baseline demographic and clinical characteristics of participants included in the immunologic comparison of humoral responses between vaccine groups. Values are reported as median (interquartile range) for continuous variables and number (percentage) for categorical variables. Anti-nucleocapsid (anti-N) antibody levels were used as a marker of prior SARS-CoV-2 infection. Comorbidities represent clinically documented chronic conditions among study participants. Unknown is number of participants from whom data are not available.

Characteristic	mRNA-1273 N = 70^*1*^	BNT162b2 N = 192^*1*^
Age median (IQR)	75 (66, 83)	76 (69, 87)
Gender		
Female	42 (60%)	111 (58%)
Male	28 (40%)	81 (42%)
Race/Ethnicity		
White Non-Hispanic	46 (66%)	156 (81%)
Black Non-Hispanic	23 (33%)	30 (16%)
Hispanic	1 (1.4%)	2 (1.0%)
Missing	0 (0%)	1 (0.5%)
Other	0 (0%)	3 (1.6%)
Dose number		
3	0 (0%)	2 (1.0%)
4	9 (13%)	35 (18%)
5	61 (87%)	155 (81%)
Days since vaccination	14.0 (14.0, 17.0)	19.0 (15.0, 21.0)
Anti-N (from prior infection)		
≤1.4 (negative)	38 (54%)	97 (51%)
>1.4 (positive)	32 (46%)	95 (49%)
Functional Status		
Independent	7 (10.4%)	24 (19.2%)
Supervision or Minimum Assist	24 (35.9%)	46 (36.8%)
Moderate or Max assist	26 (38.8%)	45 (36%)
Total assist	10 (14.9%)	10 (8.0%)
Unknown	3	67
Immunosuppressive condition or medication		
No	66 (94.3%)	147 (89.1%)
Yes	4 (5.7%)	18 (10.9%)
Unknown	0	27
Comorbidities		
Dementia	13 (18.6%)	35 (19.7%)
Cardiovascular disease	32 (45.7%)	58 (32.6%)
Pulmonary disease	23 (32.9%)	52 (29.2%)
CKD	23 (32.9%)	39 (021.9%)
Recurrent UTI	2 (2.9%)	2 (1.15)
DM	35 (50.0%)	55 (30.9%)
Hypercoagulation	13 (18.6%)	18 (10.1%)
Unknown	0	14

Serologic evidence of prior SARS-CoV-2 infection, as measured by anti-nucleocapsid antibody levels, was similar between groups. Participants in both cohorts demonstrated substantial functional dependence, consistent with a frail NHR population, although functional status data were missing for many BNT162b2 recipients. Immunosuppressive conditions were uncommon. NHRs in both groups often had one or more chronic conditions, including dementia, cardiovascular disease, pulmonary disease, chronic kidney disease or recurrent urinary tract infection, diabetes mellitus, and hypercoagulable states.

**Figure 1.**
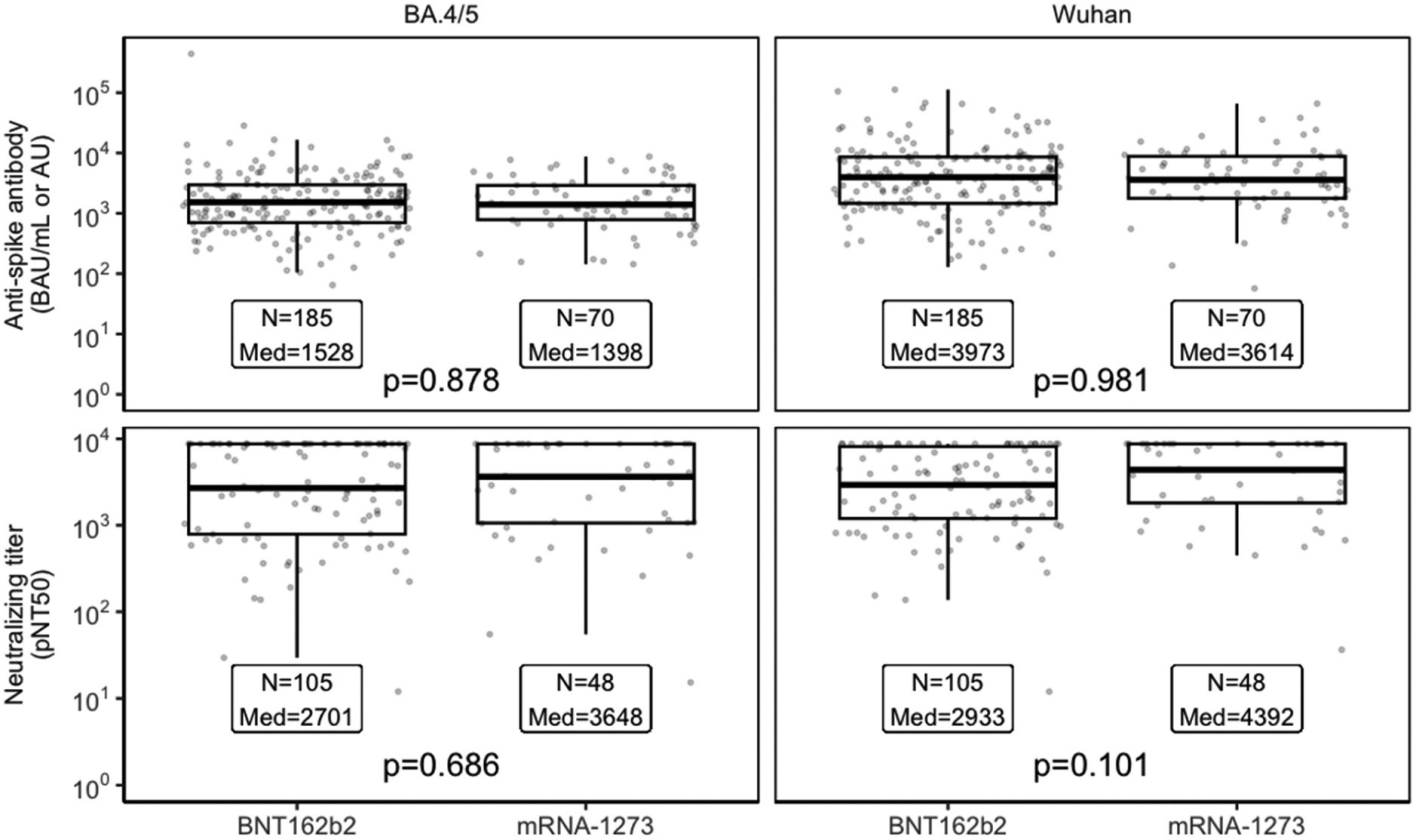
Anti-spike and neutralizing antibody responses to SARS-CoV-2 BA.4/5 and ancestral (Wuhan) strains among NHRs who received mRNA-1273 or BNT162b2 vaccines. Top panels show anti-spike IgG levels; bottom panels show neutralizing antibody titers. Samples were obtained at a median (IQR) of 14 (14–17) d for mRNA-1273 and 19 (15–21) d for BNT162b2 after vaccination.

BNT162b2 and mRNA-1273 recipients demonstrated comparable and robust anti-spike and neutralizing antibody responses against both the BA.4/5 and ancestral Wuhan SARS-CoV-2 strains (Figure 1). Median anti-spike antibody levels (BAU/ml) were slightly higher in the BNT162b2 group (1,528 vs. 1,398 for BA.4/5; 3,973 vs. 3,614 for Wuhan), but the groups were statistically similar. Similarly, neutralizing titers (pNT50) also were similar between vaccine groups, despite numerically modestly lower median values in the BNT162b2 cohort (BA.4/5: 3,648 vs. 2,701; Wuhan: 4,392 vs. 2,933). Many, though fewer than half, of the subjects elicited robust neutralization titers to one or both strains at the assay’s upper limit of detection (1:8748).

In the Table and Figure above, we excluded NHR from one facility (“Facility A”), with high enrollment among a predominantly male population, where the bivalent vaccine dose was almost exclusively mRNA-1273, and the facility’s vaccine administration schedule did not include a second monovalent booster. Therefore, we excluded Facility A from our primary analysis to avoid the potential sex and prior dose confounding with vaccine brand and facility-specific effects that could not be fully addressed within the constraints of a brief report. Sensitivity analyses that include Facility A are presented in the Supplementary Materials (Supplemental Table 1 and Supplemental Figure 1).

While the inclusion of Facility A does not materially change the overall findings, its unique composition warrants separate presentation. Including all facilities, mRNA-1273 and BNT162b2 recipients still demonstrate immune responses without any statistically significant differences (Supplemental Figure 1), and the additional 72 subjects from Facility A who received mRNA-1273 result in median neutralizing values between the vaccines being even more similar. This approach allows us to maintain clarity in the primary analysis while providing transparency and access to the full recruited cohort for readers.

## Discussion

In this immunologic evaluation of mRNA-1273 and BNT162b2 vaccines among United States NHRs, we found that both vaccines elicited comparable and robust humoral immune responses, including spike-specific binding antibodies and neutralizing titers, against both the ancestral Wuhan strain and the Omicron BA.4/5 subvariants. These findings suggest that, despite differences in formulation and dosage, the two vaccines generate similarly robust antibody responses in this frail, older adult population and are consistent with findings suggesting clinical protection.^16^ Although some studies showed a comparative advantage of mRNA-1273 over BNT162b2 in inducing modestly higher antibody levels and more durable humoral responses,^12–15^ these findings were primarily from studies conducted during following primary series or early booster doses, and prior to the emergence of Omicron and the deployment of the bivalent vaccine formulation.

Also, early in the pandemic, real-world data suggested that mRNA-1273 offered slightly greater clinical protection than BNT162b2, with studies reporting lower risks of infection and hospitalization following the primary series, particularly during the pre-Delta and Delta periods.^17–19^ However, more recent evidence from the booster era, especially during Omicron subvariant waves indicates that updated booster doses, including bivalent formulations, have narrowed these differences. Both mRNA vaccines now provide similarly strong protection against severe disease and hospitalization after booster doses.^20,21^

The absence of statistically significant differences in the neutralization and anti-spike IgG titers between recipients of mRNA-1273 and BNT162b2 in this study reinforces the functional interchangeability of these mRNA vaccine formulations with respect to short-term immune response measured in this study. However, this finding should not be interpreted as evidence of equivalence in long-term clinical effectiveness or durability of protection. This observation is particularly important in the context of booster doses for older adults, where logistical flexibility in vaccine allocation remains a public health priority. Although the two vaccines differ in mRNA content, lipid nanoparticle formulation, and dosing schedule, our findings suggest that these variables do not translate into significant disparities in antibody induction within this extensively vaccinated NH population. Despite the biological challenges posed by aging immune systems and the presence of multiple chronic conditions,^22,23^ participants in both vaccine groups mounted measurable levels of spike-specific binding antibodies and neutralizing titers against both ancestral and Omicron BA.4/5 variants. Thus, for frail individuals residing in long-term care facilities, who are very often excluded from clinical trials, mRNA vaccination remains highly immunogenic.^24–28^

Our study comes with limitations. First, our sample size is modest, which may limit the statistical power to detect small differences between vaccine groups. However, our smallest group comparisons (105 vs. 48) have sample sizes sufficient to detect a moderate effect size (Cohen’s d = 0.5) at 80% power. Secondly, we designed this study to evaluate immunogenicity, specifically binding and neutralizing antibody responses, and therefore did not assess clinical outcomes. Finally, a proportion of participants reached the upper limit of detection for the neutralization assay, which resulted in imprecision of the estimate for true differences in peak responses between groups. For this reason, we present median results, since less than half of the participants reached the upper limit of detection, and compare groups using nonparametric methods. The binding assay did not have an upper limit of detection and was similar between the two vaccines supporting the finding of relative equivalence.

## Conclusions

Both mRNA-1273 and BNT162b2 vaccines elicit similarly robust humoral immune responses in the U.S. NHRs, a population at heightened risk of severe COVID-19 outcomes. Despite formulation and dose differences, the absence of statistically significant differences in the binding and neutralizing antibody responses supports the interchangeable use of either mRNA formulation for booster vaccination in this vulnerable group.

## Supplementary Material

HVI P v M SUpfiles.docx

## Data Availability

Data are available on request to the corresponding authors.
