# Supplementary material for: Comparative immunogenicity of mRNA-1273 and BNT162b2 SARS-CoV-2 bivalent booster vaccines in US nursing home residents
Source: Hum Vaccin Immunother. 2026 Aug 4;22(1):2705661. doi: 10.1080/21645515.2026.2705661 (PMC13471111; doi:10.1080/21645515.2026.2705661)
Supplement: Supplementary file 1 [file KHVI_A_2705661_SM7651.docx]

**Appendix**

**Supplement Table and Figure**

***Supplementary Table.*** *Demographic characteristics of 32 facilities including the 72 subjects from Facility A, which were excluded from the primary analysis. Noted in bold are the key differences why this facility was included separately in supplemental data.*

| **Characteristic** | **mRNA-1273**  N = 142*^1^* | **BNT162b2**  N = 194*^1^* |
| --- | --- | --- |
| Age, median (IQR) | 75 (69, 81) | 76 (69, 87) |
| Gender |  |  |
| Female | 49 (35%) | 111 (57%) |
| Male | **93 (65%)** | **83 (43%)** |
| Race and Ethnicity |  |  |
| White Non-Hispanic | 108(76%) | 157(81%) |
| Black Non-Hispanic | 33 (23%) | 31 (16%) |
| Hispanic | 1 (0.7%) | 2 (1.0%) |
| Missing | 0 (0%) | 1 (0.5%) |
| Other | 0 (0%) | 3 (1.5%) |
|  |  |  |
| Prior doses (PS = 2) |  |  |
| 2 | 1 (0.7%) | 2 (1.0%) |
| 3 | **79 (56%)** | **36 (19%)** |
| 4 | **62 (44%)** | **156 (80%)** |
| Days since, median (IQR) | 14.0 (14.0, 15.0) | 19.0 (15.0, 21.0) |
| Facility |  |  |
| Other facilities | 70 (49%) | 192 (99%) |
| Facility A | 72 (51%) | 2 (1.0%) |
| Nucleocapsid  (N antigen) |  |  |
| <=1.4 | 76 (54%) | 97 (50%) |
| >1.4 | 66 (46%) | 97 (50%) |
| *IQR = Interquartile range (1^st^ quartile, 3^rd^ quartile)* | | |

***Supplemental*** ***Figure 1.*** *Anti-spike and neutralizing antibody responses to SARS-CoV-2 BA.4/5 and ancestral (Wuhan) strains among NHRs who received* mRNA-1273 *or* BNT162b2 *vaccines, including the large nearly all male NH. Top panels show anti-spike IgG levels; bottom panels show neutralizing antibody titers. Samples were obtained at a median (IQR) of* 14 (14–15) days for mRNA-1273 and 19 (15–21) days for BNT162b2 *after vaccination.*
